# Supplementary material for: Cerebral cortical activation and muscle performance during blood flow restriction training after ischemic stroke: A randomised functional near-infrared spectroscopy study
Source: PLoS One. 2025 Oct 9;20(10):e0334123. doi: 10.1371/journal.pone.0334123 (PMC12510505; doi:10.1371/journal.pone.0334123)
Supplement: S2 Appendix — (PDF) [file pone.0334123.s002.pdf]

连云港市第一人民医院

临床科研课题研究方案

项 目 名 称：基于 ICF 框架下血流限制联合有氧训练对卒中相关肌

少症康复效果临床研究

承 担 科 室 ：神经康复科

项目负责人：朱永刚

联 系 电 话 ：18961325963

研 究 年 限：2023 年 12 月 11 日到 2026 年 12 月 11 日

版 本 号 ：V1.0

版 本 日 期：2023 年 5 月 20 日

方案摘要

项目名称	基于 ICF 框架下血流限制联合有氧训练对卒中相关肌少症康复效果临床研究
研究目的	1、探讨血流限制联合有氧训练对脑卒中患者的肌肉表现、脑激活程度、心肺活动能力、认知功能及生活质量的影响； 2、比较有/无血流限制及不同强度的有氧训练方案的短期和长期疗效；
研究设计	随机对照设计
病例总数	60 例
病例选择	入选标准：<br>① 年龄 45-75 岁；<br>② 符合《中国急性缺血性脑卒中诊治指南 2018》的诊断标准，并经过头颅 CT 或 MRI 证实存在单侧新发脑梗死病灶；首次发病；生命体征平稳，无意识障碍；<br>③ 均合并单侧偏瘫，偏瘫侧下肢运动功能肌力≥III级<br>④ 病程 2 周-6 个月且近 1 月内病情无加重趋势；<br>⑤患者及家属知情同意并签署知情同意书；<br>⑥能配合完成所有测试的住院患者。
	排除标准：<br>① 严重认知障碍（简易精神状态量表<18 分）；<br>② 大面积皮质病变（＞大脑中动脉区域的 1/3）、严重颈动脉病变（＞90%）或脑血管狭窄（＞75%）；

	③ 有静脉血栓病史或凝血功能障碍；<br>④合并严重基础疾病、功能障碍、全身感染及恶性肿瘤等不能耐受康复训练；<br>⑤　严重的头皮皮炎。
治疗方案	干预方案为为期 4 周,每周 5 次的 MOTOmed intelligent exercise training (MOTOmed Viva 2, RECK company, Germany)的抗阻模式进行下肢训练。开始前热身 3min,每次骑行 3min,休息 1min 为 1 组,每次训练重复循环 5 组。低强度血流限制组（BFR）在 低强度训练组（LL）基础下肢训练时置于股骨近端，双侧大腿上 7cm 恒定加压，目标压强(mmHg) 200mmhg，开始训练前 5s 向袖带内充气，充气加压后开始训练。每组训练结束后，将袖带内的气体放空，组间休息的最后 5s 再向袖带内充气，再重复进行下一组训练。BFR 组、LL 组与高强度组（HL）分别以 30%、30%和 80%最大负荷重量（1RM）作为负荷强度进行下肢骑行训练。
疗效评定	有效性评价指标（主要疗效指标和次要疗效指标）：<br>主要疗效指标：在基线及 4 周后分别采用近红外脑功能成像（fNIRS）测量骑行状态下含氧血红蛋白（HbO）浓度。<br>次要疗效指标：股直肌横截面积（RFCSA）、峰力矩（PT）与下肢 Fugl-meyer (FMA-LL)。

	安全性评价指标：<br>康复治疗期间不良心血管事件的发生率。①袖带捆绑处皮肤损伤发生率：每次干预前后护士观察患者大腿近端皮肤情况并记录；②下肢深静脉血栓发生率：研究人员在患者出院当天进行超声评估。
统计方法	采用 SPSS 26.0 进行统计学分析。基线特征采用均值（标准差）、中位数（四分位数间距）或百分比表示。计量资料采用 Shapiro-Wilk 检验组水平数据的正态性及方差齐性。对于服从正态分布的数据，采用两因素方差分析以评估组/兴趣区 HbO 的主要和交互效应。采用单因素方差分析进行组间比较。相关性分析皮层 HBO 与运动表现之间的相关性。
研究期限	2023 年 12 月至 2026 年 12 月

一、研究背景

1.1 国内外研究现状

全球人口老龄化加速演进，脑血管疾病发病率随之也逐年增长。2021 年《柳叶刀》研究显示，1990-2019 脑卒中是第二大死亡原因和 第三大残疾原因[1]。卒中幸存者中超 60%的患者仍然残疾，50%的患 者存在轻偏瘫，30%的患者在没有帮助的情况下仍然无法行走[2]。骨骼肌是脑卒中残疾的主要效应器官。然而，这种残疾传统上归因于脑损伤本身，我们较少关注到肌肉组织的结构,代谢和功能方面。肌肉组织的结构适应性变化早在卒中后 4 小时开始，且非受累肢体在中风后 1 周内也会出现肌无力[3]，主要表现为单侧或双侧的肌肉含量下降、肌肉力量和身体功能减退，从而增加病人如跌倒、骨折、残疾甚至死亡的风险[4]。目前，还没有特定的药物被批准用于治疗卒中后肌肉无力，因此运动疗法仍然是最有效策略。

血流限制疗法（BFRT）又称加压疗法，最早在日本推行，一些科学家认为它是“最先进”的锻炼方式。BFRT 提供了一种新的训练方法来改善肌肉减少[5]，其模拟高强度负荷，以低负荷运动增加肌肉力量和肌容积，达到与高负荷训练相似的增益效果[6]。具体实施办法是在肢体近心端使用绑带对骨骼肌施加压力，阻断组织的静脉回流，不阻断或部分阻断动脉血流的条件下进行运动。BFRT 期间涉及复杂的机制，目前，所提出的机制主要包括血管和相关激素的反应，例如生长因子，组织缺氧相关因子以及肌肉纤维以及肌肉卫星细胞的募集[7]，这些机制有助于骨骼肌合成的正平衡，从而减轻肌肉虚弱症状[8]。

血流限制疗法被广泛应用于年老体弱者和肌肉骨骼损伤后恢复者及运动员，然而，当前对于脑卒中患者的应用的证据很少。卒中后继发的肌肉骨骼问题限制活动及社会参与，应充分重视卒中后肌肉萎缩无力、行走能力受损问题。

先前的神经病学研究证据表明，BFRT 可改善多发性硬化症和不完全脊髓损伤患者的肌肉力量、平衡、步行和认知功能[9-11]。最近的一项研究表明，BFRT 训练改善了缺血性中风患者的脑源性神经营养因子（BDNF）和血管内皮生长因子（VEGF）以及疲劳感知能力[12]。 尽管低强度抗阻力训练对脑卒中患者的平衡、肌力、行走能力均有益 处 ，但有氧结合相比阻力运动更适合长期使用以发展肌肉容积和力 量。此外，一项系统评价纳入了 10 项临床对照试验，研究有氧训练 对脑卒中受试者的治疗效果，发现整体认知能力有显著提高，并在一 定程度上对某些认知领域（主要是记忆力、注意力和视觉空间能力） 产生了积极影响[13]。

目前，还没有临床试验研究过有氧训练结合 BFRT 对老年脑卒中的积极影响。《国际功能、残疾和健康分类》（ICF）依据的是在身体、个体和社会 3 个层面的健康状态。本研究将在 ICF 框架下应用血流限制联合有氧训练探究其对卒中的康复效果。

1.2 研究的目的、意义

有氧训练和血流限制疗法均可以改善骨骼肌的容积和力量，但其对脑卒中继发的肌肉虚弱人群的影响尚不清楚。我们试图探究（1）血流限制联合骑行康复任务在脑卒中患者脑激活模式的相关变化。

（即刻效应）。（2）干预后，脑卒中患者脑激活变化及运动机能表现特征研究。

理论意义：（1）本研究围绕脑卒中患者展开干预，填补了当前此疾病运动疗法文献的空白，尤其是对于脑卒中患侧运动的中枢和外周肌肉激活程度。（2）我们探究一种新兴运动疗法——血流限制联合有氧训练的康复优势，为进一步推荐或拒绝使用血流限制疗法提供了理论依据。

实践意义：（1）本研究将明确提出了关于卒中后肌肉无力的干预方案和强度方面的建议，可推动以 BFRT 为工具的康复手段在临床实践中的使用，将有广阔的应用前景。（2）BFRT 可能是一种经济、省时的方法，可以为行动不便的脑卒中患者制定更安全、可行且经济有效的训练计划。

二、研究目的

1. 主要目的：探讨血流限制联合有氧训练对卒中患者的脑激活程度，肌肉表现（肌肉横截面积、力量、表现）影响。

2. 次要目的：比较有/无血流限制及不同强度的有氧训练方案的短期和长期疗效；

三、 研究设计类型、原则与试验步骤

1. 研究设计

本研究为随机对照设计；选取在我院神经内、外科和康复科住院的脑梗死患者若干例。脑梗死诊断需符合《中国急性缺血性脑卒中诊治指南 2018》的诊断标准，并经过头颅 CT 或 MRI 证实存在单侧新发脑梗死病灶；首次发病；生命体征平稳，无意识障碍。

2. 随机对照方法

按照性别与 1RM 确定的阻值(3-8;9-14;15-20)进行分层的随机的方法将入选的患者平均分为 3 组，即 BFR 组/LI 组/HI 组。由计算机随机程序产生随机数字表，数字对应相应字母，将分组方案依次隐藏在不透光的信封里，所有的随机信封由一名不参与课题操作和数据分析的研究者保存。在脑卒中患者的基线评价完成，患者及家属签署知情同意书完成后，研究者根据脑卒中患者的入组顺序选取并拆开一个信封，从而取得患者随机号，得到随机号的患者被带到研究测试者处。包含随机号码的信封有 3 套，为避免研究者及相关研究人员随意调整患者的分组情况，随机分组具体方案及患者编号对测试者及治疗师不设盲，对试验评价者及数据统计者设盲。

3.实验样本量

根据以往使用 fNIRS 检测脑卒中人群皮层重组的研究[21]，我们使用 SPSS 15.0 进行看功率分析和先验样本量估计。我们设定 α 误差为 0.05，β 为 0.10（功率水平为 0.90）。根据分析，至少需要 57 名患者，假设由于脑卒中患者在运动训练范式中运动伪影过多而导致的辍学率为 15%，则每组至少需要 22 名患者。

四、病例选择

1．入选标准

①年龄 45-75 岁；②首次皮层下缺血性卒中发作并伴有偏瘫，发作后 2 周-6 个月；③ 单侧脑室上病变；④患侧下肢主要屈伸肌群≥3 级（徒手肌力测试）；⑤能够配合完成 fNIRS 任务的住院患者；⑥

患者及家属知情同意并签署知情同意书。

2．排除标准

①严重认知功能障碍，简易精神状态量表（MMSE）得分＜18；②大面积皮质病变（＞大脑中动脉区域的 1/3）、严重颈动脉病变（＞90%）或脑血管狭窄（＞75%）；③ 全身不稳定；④严重头皮皮炎。

3．终止研究标准

严重的不良事件包括脑卒中二次发作或短暂性脑缺血、急性心肌梗死、需临床干预的心律失常、骨折、肺栓塞和死亡。非严重的不良事件包括跌倒、剧烈疼痛或肌肉酸痛、血压过高或不升反降、无症状心肌缺血和头晕。发生的不良事件将立即记载并告知给负责的医生，同时做相应急救措施。

五、研究方法与技术路线

1. 干预方案

应用 MOTOmed intelligent exercise training (MOTOmed Viva 2, RECK company, Germany)的抗阻模式进行训练，BFR 与 LL 组均以 30%1RM ，HL 组以 80%1RM 进行 3min 热身和 20min（4min[3min 踩车+1min 休息]为一组*5）的抗阻踩车，5 次/周，持续 4 周。BFR 组在 CON 组基础上，使用宽度为 7cm 的 pneumatic cuffs (B STRONG，美国)在患侧下肢近端（腹股沟皱襞）进行加压。根据健康受试者的研究，引起最大脑激活的安全压力为 250mmHg；考虑到脑卒中患者循环可能受阻，故我们将压力值设定在其 80%，即 200mmHg。我们在开始训练前 10s 向袖带内充气，每组训练结束后，将袖带内的气体放空，组间休息的最后 10s 再向袖带内充气。值得注意的是，我们采用间歇加压法（[3 分钟加压踩踏+1 分钟释压休息]*5）。因为其相比

连续性加压能更有效地诱导肌肉适应刺激。除此之外，所有受试者在干预期间都接受了常规药物治疗，物理治疗和职业治疗。注意：训练过程一名治疗师全程监督。训练前辅助患者调整功率自行车座椅及把手高度，确保患者骑行过程中的舒适程度，告知患者的具体训练方案及时间间隔情况；嘱患者在骑行过程中尽量保持上身的直立，减少骑行过程中上身的摆动代偿，保证患者的训练强度；同时训练过程中密切关注患者疲劳程度及身体不适的主观感受等情况，如有异常发生，为保证患者安全立即停止试验，并告知患者主管医生，分析患者是否需要完善相关检查以便明确出现问题的原因。

2.技术路线图

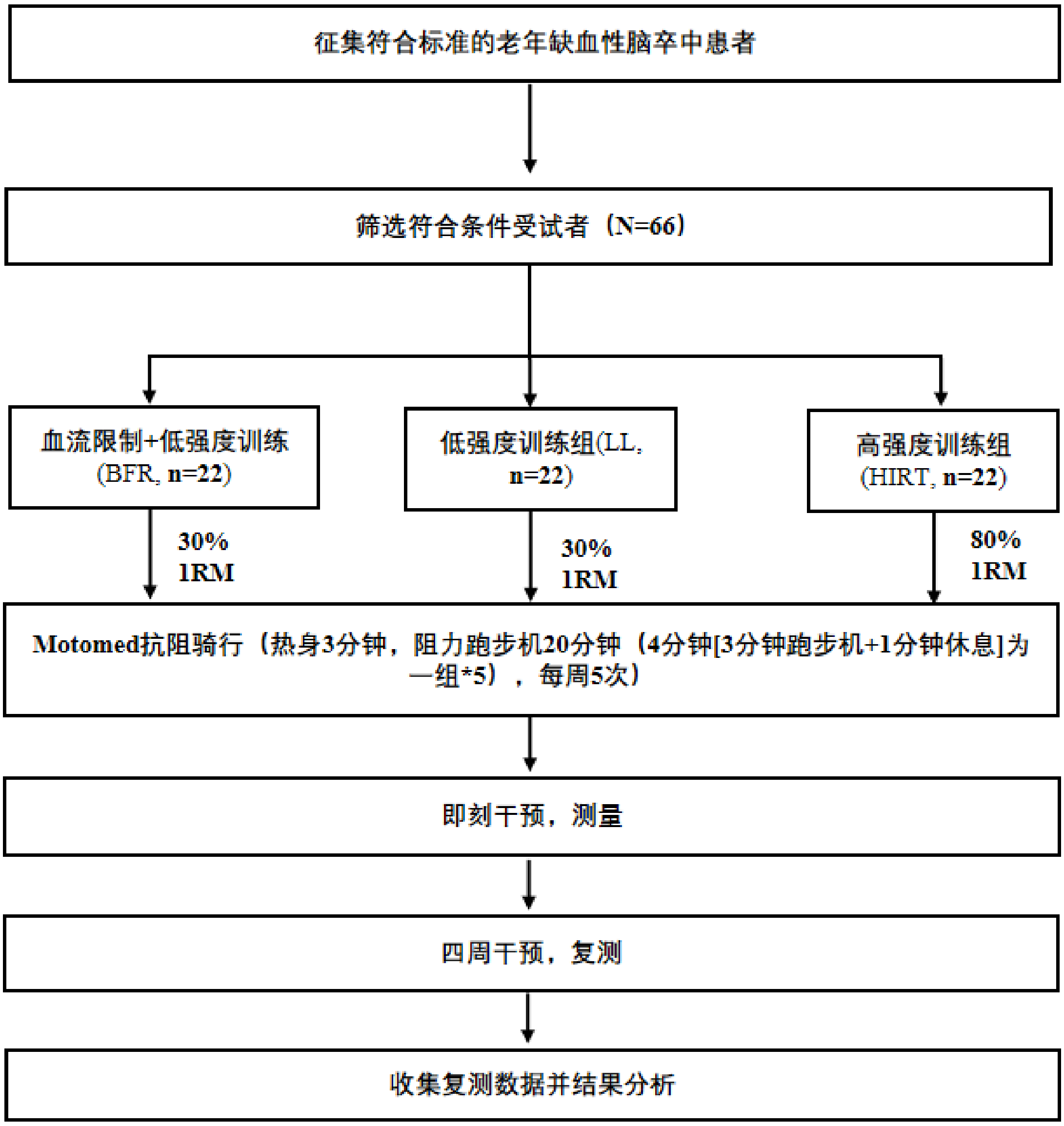

六、观察项目与检测时点

1、　一般资料

对于纳入患者入院当天收集患者基本信息和人体测量参数，包括性别、年龄、身高、体重、吸烟饮酒史、脑卒中类型、偏瘫侧别、脑卒中持续时间、基础疾病；其余指标在治疗前评估得分，并在治疗 4 周后（治疗后）再次评估。

2、脑激活水平

使用了多通道 fNIRS 设备（型号 NirSmart-6000，慧创，中国丹

阳）以 11 Hz 采样率捕捉脑血流动力学信号，并计算了相应的血红蛋白和脱氧血红蛋白密度。后续的研究分析中我们依托 HBO 推断大脑皮层激活程度，这主要是由于 HBO 相比 Hb 拥有更高的信噪比和更好的测量信度。采集帽上有 18 个光源和 17 个探测器，共有 35 个通道。探头覆盖的区域包括双侧背外侧前额叶(DLPFC)、初级运动皮层(M1)、前运动和辅助运动皮层（PMC-SMA）。

研究者会提前与患者沟通 fNIRS 评估流程。参与者保持清醒，安静的坐在扶手椅上，避免不必要的身体活动。采用 Block 设计（休息[50 秒]后 5 个周期：踩车[20 秒]；休息[30 秒]）。评估前研究者根据统计学家事先确定的阻力值（30%1RM 或 80%1RM）设定每个受试者的踩车阻力，根据组别使用或不使用血流限制带，休息期间释放血流限制带的压力。所有参与者在实验前练习任务 3 到 5 分钟。本研究收集了踩车相关的 fNIRS 数据，受试者均进行两次 fNIRS 扫描程序：在没有任何干预的下获得初始数据，以及四周干预后的数据。

3、运动能力：

肌肉形态：肌骨超声（柯尼卡美能达，中国）选择 L18-4 线阵探头、肌骨模式膝关节部位。患者取卧位，膝下垫滚轴保持屈膝约 30°，探头短轴垂直皮肤表面测量髌骨上极 10cm 处股直肌厚度、股直肌横截面积，使用较多耦合剂并减少加压，降低对软组织的压迫[7]。

伸膝力量：等速肌力测试系统（广州一康，中国）测量伸膝峰力矩(peak torque，PT).患者取坐位，配置等速膝关节配件，热身 5 次，60°/s 角速度下完成最大伸膝屈膝向心运动，重复 5 次。记录最大伸

膝峰力矩，单位为 N·m, 代表肌肉收缩的最大肌力。

运动功能：采用 Fugl-Meyer 运动功能评定量表(Fugl-meyer motor assessment of lower limbs, FMA-LL) 对干预前、后下肢运动功能进行评估，分为仰卧位下肢反射运动、屈肌共同运动、伸肌抗阻运动；坐位的联合共同运动；站立位分离运 动；坐位的正常反射及仰卧位协调/速度。共 7 大项，总计 34 小项，每一小项为 1 分，总计 34 分，得分越高，说明下肢功能恢复越好。

七、疗效评定标准

疗效评价指标：1、脑激活水平：脑皮质局部血红蛋白浓度。2. 肌肉表现：超声股直肌横截面积表明肌量变化，等速肌力峰力矩表明肌肉爆发力；3、躯体运动功能： 下肢 Fugl-Meyer 量表；

安全性评价：康复治疗期间不良心血管事件的发生率。1)袖带捆绑处皮肤损伤发生率：每次干预前后护士观察患者大腿近端皮肤情况并记录；2)下肢深静脉血栓 发生率：研究人员在患者出院当天进行超声评估。

八、不良事件的观察

严重的不良事件包括脑卒中二次发作或短暂性脑缺血、急性心肌梗死、需临床干预的心律失常、骨折、肺栓塞和死亡。非严重的不良事件包括跌倒、剧烈疼痛或肌肉酸痛、血压过高或不升反降、无症状心肌缺血和头晕。发生的不良事件将立即记载并告知给负责的医生，同时做相应急救措施。

九、研究的质量控制与质量保证

本课题组成员具备熟练的技术能力可保证本研究方案顺利实施。课题组所在的连云港市第一人民医院神经康复科具备本实验所需的

主要设备和条件，可作为稳固的技术依托，具备充足的脑卒中病源保证试验可以连续开展。各项功能评估采用盲法，保证评估治疗的科学性严谨性，患者及家属接受相关知识注意事项告知，保证受试者依从性。课题组成员年轻优秀：课题组成员结构合理，涵盖了本课题所需的各种技能，团结协作，各司其职，能确保工作的顺利开展。

十、数据安全监查

临床研究将根据风险大小制定相应的数据安全监察计划。所有不良事件均详细记录，恰当处理并追踪直到妥善解决或病情稳定，按照规定及时向伦理委员会、主管部门、申办者和药品监督管理部门报告严重不良事件与非预期事件等；主要研究者定期对所有不良事件进行累积性回顾，必要时召开研究者会议评估研究的风险与受益；双盲试验必要时可以进行紧急揭盲，以确保受试者安全与权益；大于最小风险的研究将安排独立的数据监查员对研究数据进行监查，高风险研究将建立独立的数据安全监察委员会对累积的安全性数据以及有效性数据进行监查，以做出研究是否继续进行的建议。

十一、统计学处理

数据特征采用均值（标准差）、中位数（四分位数间距）或百分比表示。计量资料采用 Shapiro-Wilk 检验和 Levene's 检验数据的正态性和方差齐性，对于符合正态分布的数据使用单因素方差分析检验差异性，不符则使用 Kruskal-Wallis 秩和检验。计数资料使用卡方检验差异性。采用两因素方差分析以评估组，兴趣区 HbOmean 的主要和交互效应，如果显著，则遵循 LSD 事后检验。所有数据在 SPSS26.0 中分析，显著性设定为 $P<0.05$。使用皮尔逊相关分析评估皮层 HBO 中与运动表现之间的相关性。

十二、临床研究的伦理学

临床研究将遵循世界医学大会《赫尔辛基宣言》等相关规定。在研究开始之前，由伦理委员会批准该试验方案后才实施临床研究。每一位受试者入选本研究前，研究者有责任向受试者或其代理人完整、全面地介绍本研究的目的、程序和可能的风险，并签署书面知情同意书，应让受试者知道他们有权随时退出本研究，知情同意中应作为临床研究文件保留备查。研究过程中将保护受试者的个人隐私与数据机密性。

十三、研究进度

1、 2023 年 12 月— 2023 年 06 月：受试者筛选、分组

2、 2024 年 07 月— 2024 年 12 月：干预实施，数据采集

3、 2025 年 1 月— 2025 年 06 月: 干预实施，数据采集

4、 2025 年 07 月— 2025 年 12 月: 临床试验数据收集与整理

5、 2026 年 1 月— 2026 年 06 月: 完成所有病例的临床试验

6、 2026 年 07 月— 2026 年 12 月：统计学分析，撰写论文，申报科技进步奖

十四、项目组成员

<table>
<tr><td rowspan="2">项目负责人</td><td>姓 名</td><td>朱永刚</td><td>性别</td><td>男</td><td>出 生 年 月</td><td colspan="2">1976 年 3 月 3 日</td></tr>
<tr><td>专业技术职称</td><td>正高</td><td>学位</td><td>医学硕士</td><td>专 业</td><td colspan="2">临床医学</td></tr>
<tr><td rowspan="2">项目组</td><td>总人数</td><td>高级</td><td>中级</td><td>初级</td><td>博士后</td><td>博士</td><td>硕士</td><td>辅助人员</td></tr>
<tr><td>6</td><td>1</td><td>3</td><td>3</td><td></td><td>1</td><td>3</td><td></td></tr>
<tr><td rowspan="3">项目组主要成员</td><td>姓名</td><td>年龄</td><td>专业</td><td>技 术 职称</td><td>任务分工</td><td>是否培训</td><td>培训时间</td></tr>
<tr><td>朱永刚</td><td>47</td><td>临床医学</td><td>正高</td><td>选题与审稿</td><td>是</td><td>2023.08-2023.10</td></tr>
<tr><td>白昕予</td><td>41</td><td>临床医学</td><td>中级</td><td>设计与论文撰写</td><td>是</td><td>2023.08-2023.10</td></tr>
</table>

	张云云	27	康复医学与理疗学	初级	数据分析、论文撰写	是	2023.08-2023.10
	曹伟伟	35	康复治疗学	中级	受试者招募	是	2023.08-2023.10
	谢阳	29	康复治疗学	中级	数据收集	是	2023.08-2023.10
	于洁	26	康复治疗学	初级	试验实施	是	2023.08-2023.10
	江叶	28	康复治疗学	初级	试验实施	是	2023.08-2023.10

十五、参考文献

[1] Global, regional, and national burden of stroke and its risk factors, 1990-2019: a systematic analysis for the Global Burden of Disease Study 2019 [J]. Lancet Neurol, 2021, 20(10): 795-820.

[2] Kelly-Hayes M, Beiser A, Kase CS, et al. The influence of gender and age on disability following ischemic stroke: the Framingham study [J]. J Stroke Cerebrovasc Dis, 2003, 12(3): 119-126.

[3] Harris ML, Polkey MI, Bath PM, et al. Quadriceps muscle weakness following acute hemiplegic stroke [J]. Clin Rehabil, 2001, 15(3): 274-281.

[4] Cruz-Jentoft AJ, Bahat G, Bauer J, et al. Sarcopenia: revised European consensus on definition and diagnosis [J]. Age Ageing, 2019, 48(1): 16-31.

[5] Liu Q-Q, Xie W-Q, Luo Y-X, et al. High Intensity Interval Training: A Potential Method for Treating Sarcopenia [J]. Clin Interv Aging, 2022, 17: 857-872.

[6] Chang H, Yan J, Lu G, et al. Muscle strength adaptation between high-load resistance training versus low-load blood flow restriction training with different cuff pressure characteristics: a systematic review and meta-analysis [J]. Front Physiol, 2023, 14: 1244292.

[7] Davids CJ, Roberts LA, Bjørnsen T, et al. Where Does Blood Flow Restriction Fit in the Toolbox of Athletic Development? A Narrative Review of the Proposed Mechanisms and Potential Applications [J]. Sports Med, 2023.

[8] Zhang X-Z, Xie W-Q, Chen L, et al. Blood Flow Restriction Training for the

Intervention of Sarcopenia: Current Stage and Future Perspective [J]. Front Med (Lausanne), 2022, 9: 894996.

[9] Lamberti N, Straudi S, Donadi M, et al. Effectiveness of blood flow-restricted slow walking on mobility in severe multiple sclerosis: A pilot randomized trial [J]. Scand J Med Sci Sports, 2020, 30(10): 1999-2009.

[10] Gorgey AS, Timmons MK, Dolbow DR, et al. Electrical stimulation and blood flow restriction increase wrist extensor cross-sectional area and flow meditated dilatation following spinal cord injury [J]. Eur J Appl Physiol, 2016, 116(6): 1231-1244.

[11] Freitas EDS, Miller RM, Heishman AD, et al. The perceptual responses of individuals with multiple sclerosis to blood flow restriction versus traditional resistance exercise [J]. Physiol Behav, 2021, 229: 113219.

[12] Du X, Chen W, Zhan N, et al. The effects of low-intensity resistance training with or without blood flow restriction on serum BDNF, VEGF and perception in patients with post-stroke depression [J]. Neuro Endocrinol Lett, 2021, 42(4): 229-235.

[13] Zheng G, Zhou W, Xia R, et al. Aerobic Exercises for Cognition Rehabilitation following Stroke: A Systematic Review [J]. J Stroke Cerebrovasc Dis, 2016, 25(11): 2780-2789.

Lianyungang First People's Hospital

Clinical Research Project Proposal

Project Title: Clinical Research on the Rehabilitation Effect of Blood Flow Restriction Combined with Aerobic Training on Stroke – related muscle weakness Based on the ICF Framework

Department in Charge: Neurological Rehabilitation Department

Project Leader: Zhu Yonggang

Contact Number: 18961325963

Research Duration: December 11, 2023, to December 11, 2026

Version Number: V1.0

Version Date: May 20, 2023

Abstract

Project Title	Clinical Research on the Rehabilitation Effect of Blood Flow Restriction Combined with Aerobic Training on Stroke related muscle weakness Based on the ICF Framework
Research Objectives	1. To explore the effects of blood flow restriction combined with aerobic training on muscle performance, brain activation, cardiopulmonary function, cognitive function, and quality of life in stroke patients; 2. To compare the short-term and long-term efficacy of aerobic training with and without blood flow restriction and different intensities of aerobic training
Research Design	Randomized controlled design
Total Number of Cases	60 cases
Case Selection	Inclusion Criteria: ① Age 45-75 years; ② Diagnosis of acute ischemic stroke according to the "Chinese Guidelines for the Diagnosis and Treatment of Acute Ischemic Stroke 2018," confirmed by CT or MRI with unilateral new cerebral infarction; first episode; stable vital signs, no

	consciousness disorder; ③ Unilateral hemiplegia, with muscle strength of the affected lower limb ≥ III; ④ Disease duration of 2 weeks to 6 months, with no worsening trend in the past month; ⑤ Patients and their families are informed and have signed the informed consent form; ⑥ Inpatients who can cooperate to complete all tests.
	Exclusion Criteria: ① Severe cognitive impairment (MMSE score < 18); ② Large cortical lesions (>1/3 of the middle cerebral artery area), severe carotid artery disease (>90%), or cerebrovascular stenosis (>75%); ③ History of venous thrombosis or coagulation dysfunction; ④ Severe underlying diseases, functional impairment, systemic infection, or malignant tumors that cannot tolerate rehabilitation training; ⑤ Severe scalp dermatitis.
Treatment Plan	The intervention plan involves 4 weeks of MOTOmed intelligent exercise training (MOTOmed Viva 2, RECK company, Germany) in resistance mode for lower limb training, 5 times per week. Warm-up for 3 minutes before starting, each session consists of 3 minutes of cycling, 1 minute of rest, repeated for 5 cycles. The low-intensity blood flow restriction group (BFR) applies constant pressure of 200 mmHg to the proximal femur, 7 cm above

	the thigh, during low-intensity training. The pressure is applied 5 seconds before training and released after each cycle. The BFR group, low-intensity group (LL), and high-intensity group (HL) perform lower limb cycling training at 30%, 30%, and 80% of the maximum load weight (1RM), respectively.
Efficacy Evaluation	Primary efficacy indicator: Near-infrared brain function imaging (fNIRS) to measure oxygenated hemoglobin (HbO) concentration during cycling at baseline and after 4 weeks. Secondary efficacy indicators: Rectus femoris cross-sectional area (RFCSA), peak torque (PT), and Fugl-Meyer Assessment for lower limbs (FMA-LL).
	Safety evaluation indicators: Incidence of adverse cardiovascular events during rehabilitation. ① Incidence of skin damage at the cuff site: Nurses observe and record the skin condition of the patient's proximal thigh before and after each intervention; ② Incidence of deep vein thrombosis: Ultrasound evaluation by researchers on the day of discharge.
Statistical Methods	SPSS 26.0 will be used for statistical analysis. Baseline characteristics will be expressed as mean (standard deviation), median (interquartile range), or percentage. The Shapiro-Wilk test will be used to test the normality and

	homogeneity of variance of the data. For normally distributed data, two-way ANOVA will be used to assess the main and interaction effects of group/region of interest on HbO. One-way ANOVA will be used for between-group comparisons. Pearson correlation analysis will be used to assess the correlation between cortical HbO and motor performance.
Research Duration	December 2023 to December 2026

I. Research Background

1.1 Current Research Status at Home and Abroad

The global population is aging rapidly, and the incidence of cerebrovascular diseases is increasing year by year. A 2021 study in *The Lancet* showed that stroke was the second leading cause of death and the third leading cause of disability from 1990 to 2019 [1]. Over 60% of stroke survivors remain disabled, 50% have mild hemiplegia, and 30% are unable to walk without assistance [2]. Skeletal muscle is the main effector organ of stroke-related disability. However, this disability is traditionally attributed to brain injury itself, with less attention paid to the structural, metabolic, and functional aspects of muscle tissue. Structural adaptive changes in muscle tissue begin as early as 4 hours after a stroke, and muscle weakness can occur in the non-affected limb within 1 week after a stroke [3], mainly manifested as a decrease in muscle mass, muscle strength, and physical function, increasing the risk of falls, fractures, disability, and even death [4]. Currently, no specific drugs have been approved for the treatment of post-stroke muscle weakness, so exercise therapy remains the most effective strategy.

Blood flow restriction therapy (BFRT), also known as occlusion training, was first introduced in Japan, and some scientists consider it the "most advanced" form of exercise. BFRT provides a new training method to improve muscle loss [5], simulating high-intensity load with low-load exercise to increase muscle strength and volume, achieving similar gains to high-load training [6]. The specific implementation involves applying pressure to the skeletal muscle near the proximal end of the limb using a band to block venous return without blocking or partially blocking arterial blood flow during exercise. BFRT involves complex mechanisms, including vascular and hormonal responses, such as growth factors,

hypoxia-related factors, and muscle fiber and satellite cell recruitment [7], which help maintain a positive balance in skeletal muscle synthesis, thereby alleviating muscle weakness [8]. BFRT is widely used in the elderly, those recovering from musculoskeletal injuries, and athletes. However, there is little evidence of its application in stroke patients. Post-stroke musculoskeletal problems limit activity and social participation, and attention should be paid to post-stroke muscle atrophy, weakness, and impaired walking ability.

Previous neurological research evidence suggests that BFRT can improve muscle strength, balance, walking, and cognitive function in patients with multiple sclerosis and incomplete spinal cord injury [9-11]. A recent study showed that BFRT training improved brain-derived neurotrophic factor (BDNF) and vascular endothelial growth factor (VEGF) levels, as well as fatigue perception, in ischemic stroke patients [12]. Although low-intensity resistance training is beneficial for balance, muscle strength, and walking ability in stroke patients, aerobic training combined with resistance exercise is more suitable for long-term use to develop muscle volume and strength. Additionally, a systematic review of 10 clinical controlled trials found that aerobic training significantly improved overall cognitive ability and had a positive impact on certain cognitive domains (mainly memory, attention, and visuospatial ability) in stroke patients [13].

Currently, no clinical trials have studied the positive effects of aerobic training combined with BFRT on elderly stroke patients. The *International Classification of Functioning, Disability, and Health* (ICF) is based on health status at the body, individual, and social levels. This study will explore the rehabilitation effects of blood flow restriction combined with aerobic training on stroke within the ICF framework.

1.2 Research Objectives and Significance

Both aerobic training and blood flow restriction therapy can improve muscle volume and strength, but their effects on stroke-related muscle weakness are unclear. We aim to explore (1) the immediate effects of blood flow restriction combined with cycling rehabilitation on brain activation patterns in stroke patients; (2) changes in brain activation and motor performance in stroke patients after intervention.

Theoretical significance: (1) This study focuses on stroke patients, filling a gap in the current literature on exercise therapy for this condition, especially regarding central and peripheral muscle activation in stroke-affected limbs. (2) We explore the advantages of a new exercise therapy—blood flow restriction combined with aerobic training—providing a theoretical basis for recommending or rejecting the use of blood flow restriction therapy.

Practical significance: (1) This study will provide clear recommendations for intervention plans and intensity for post-stroke muscle weakness, promoting the use of BFRT as a rehabilitation tool in clinical practice, with broad application prospects. (2) BFRT may be an economical and time-saving method, offering a safer, feasible, and cost-effective training plan for stroke patients with limited mobility.

II. Research Objectives

1. Primary Objective: To explore the effects of blood flow restriction combined with aerobic training on brain activation, muscle performance (muscle cross-sectional area, strength, performance) in stroke patients.

2. Secondary Objective: To compare the short-term and long-term efficacy of aerobic training with and without blood flow restriction and different intensities of aerobic training.

III. Research Design, Principles, and Experimental Steps

1. Research Design

This study is a randomized controlled design; patients with cerebral infarction admitted to the neurology, neurosurgery, and rehabilitation departments of our hospital will be selected. The diagnosis of cerebral infarction must meet the diagnostic criteria of the "Chinese Guidelines for the Diagnosis and Treatment of Acute Ischemic Stroke 2018," confirmed by CT or MRI with unilateral new cerebral infarction; first episode; stable vital signs, no consciousness disorder.

2. Randomization Method

Patients will be stratified by gender and 1RM-determined resistance values (3-8; 9-14; 15-20) and randomly divided into three groups: BFR group, LL group, and HL group. A computer-generated random number table will assign numbers to corresponding letters, and the grouping scheme will be hidden in opaque envelopes. All random envelopes will be kept by a researcher not involved in the operation and data analysis. After baseline evaluation and informed consent, the researcher will select and open an envelope to obtain the patient's random number, and the patient will be assigned to the corresponding group. To prevent researchers from adjusting the grouping, the randomization scheme and patient numbers will be blinded to the evaluators and statisticians.

3. Sample Size

Based on previous studies using fNIRS to detect cortical reorganization in stroke patients [21], we used SPSS 15.0 for power analysis and a priori sample size estimation. We set α error at 0.05 and β at 0.10 (power level of 0.90). The analysis showed that at least 57 patients are needed, assuming a 15% dropout rate due to excessive motion artifacts in stroke patients during exercise training paradigms, requiring at least 22 patients per group.

IV. Case Selection

1. Inclusion Criteria

① Age 45-75 years; ② First episode of subcortical ischemic stroke with hemiplegia, 2 weeks to 6 months after onset; ③ Unilateral supratentorial lesion; ④ Muscle strength of the main flexor and extensor muscles of the affected lower limb ≥ III (manual muscle testing); ⑤ Inpatients who can cooperate to complete fNIRS tasks; ⑥ Patients and their families are informed and have signed the informed consent form.

2. Exclusion Criteria

① Severe cognitive dysfunction, MMSE score < 18; ② Large cortical lesions (>1/3 of the middle cerebral artery area), severe carotid artery disease (>90%), or cerebrovascular stenosis (>75%); ③ Systemic instability; ④ Severe scalp dermatitis.

3. Termination Criteria

Serious adverse events include secondary stroke or transient ischemic attack, acute myocardial infarction, clinically significant arrhythmia, fracture, pulmonary embolism, and death. Non-serious adverse events include falls, severe pain or muscle soreness, excessively high or low blood pressure, asymptomatic myocardial ischemia, and dizziness. Adverse events will be recorded immediately and reported to the responsible physician, with appropriate emergency measures taken.

V. Research Methods and Technical Route

1. Intervention Plan

MOTOmed intelligent exercise training (MOTOmed Viva 2, RECK company, Germany) will be used in resistance mode. The BFR and LL groups will perform 3 minutes of warm-up and 20 minutes (4 minutes [3 minutes cycling + 1 minute rest] × 5) of resistance cycling at 30% 1RM, 5 times per week for 4 weeks. The BFR group will use 7 cm wide pneumatic cuffs (B STRONG, USA) to apply pressure to the proximal end of the affected lower limb (inguinal fold). Based on healthy subjects, the safe pressure for maximum brain activation is 250 mmHg;

considering possible circulatory obstruction in stroke patients, we set the pressure at 80% of this value, i.e., 200 mmHg. The cuff will be inflated 10 seconds before training and deflated after each cycle, with the cuff reinflated in the last 10 seconds of rest. Notably, we use intermittent pressure application ([3 minutes of pressurized cycling + 1 minute of pressure release] × 5), as it is more effective in inducing muscle adaptation to stimulation. Additionally, all subjects will receive conventional drug therapy, physical therapy, and occupational therapy during the intervention. Note: A therapist will supervise the entire training process. Before training, the therapist will assist the patient in adjusting the seat and handle height of the exercise bike to ensure comfort during cycling, inform the patient of the specific training plan and time intervals, instruct the patient to maintain an upright posture during cycling to reduce compensatory movements, ensure training intensity, and closely monitor the patient's fatigue level and subjective feelings of discomfort during training. If any abnormalities occur, the trial will be stopped immediately for patient safety, and the patient's attending physician will be informed to determine if further tests are needed to clarify the cause of the problem.

2. Technical Route Diagram

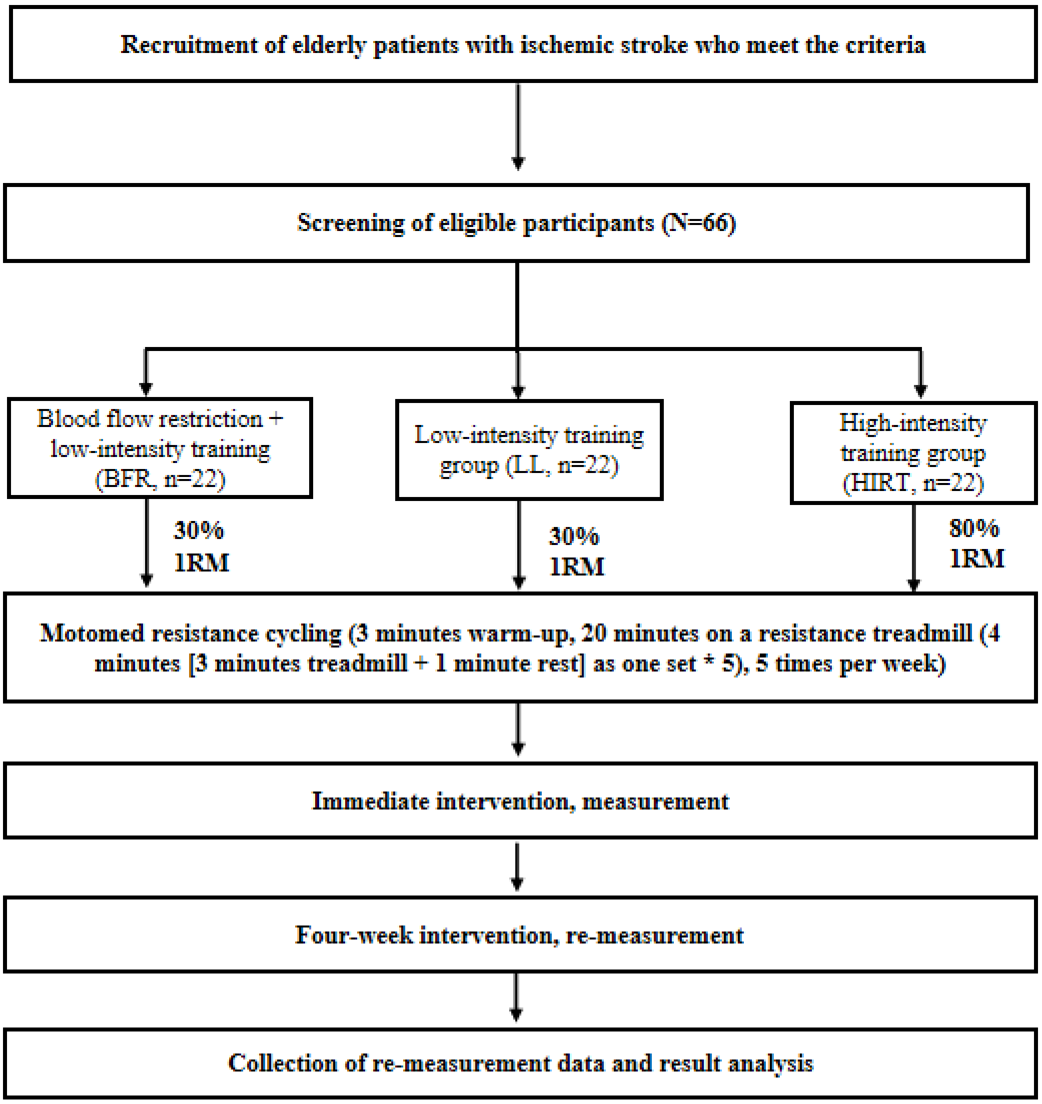

VI. Observation Items and Detection Time Points

1. General Information

On the day of admission, basic information and anthropometric parameters of the included patients will be collected, including gender, age, height, weight, smoking and drinking history, stroke type, hemiplegic side, stroke duration, and underlying diseases; other indicators will be assessed before treatment and reassessed after 4 weeks of treatment.

2. Brain Activation Level

A multi-channel fNIRS device (model NirSmart-6000, HuiChuang, Danyang, China) will be used to capture cerebral hemodynamic signals at a sampling rate of 11 Hz, and the corresponding hemoglobin and deoxyhemoglobin densities will be calculated. In subsequent analyses, we will rely on HbO to infer cortical activation, as HbO has a higher signal-to-noise ratio and better measurement reliability than Hb. The cap has 18 light sources and 17 detectors, totaling 35 channels. The probe covers the bilateral dorsolateral prefrontal cortex (DLPFC), primary motor cortex (M1), and premotor and supplementary motor cortex (PMC-SMA).

The researcher will communicate the fNIRS assessment process with the patient in advance. Participants will remain awake and sit quietly in an armchair, avoiding unnecessary body movements. A block design (rest [50 seconds] followed by 5 cycles: cycling [20 seconds]; rest [30 seconds]) will be used. Before the assessment, the researcher will set the cycling resistance for each subject based on the resistance value (30% 1RM or 80% 1RM) determined by the statistician in advance, and use or not use the blood flow restriction band according to the group, releasing the pressure of the blood flow restriction band during rest. All participants will practice the task for 3 to 5 minutes before the experiment. This study will collect fNIRS data related to cycling, and all subjects will undergo two fNIRS scanning procedures: initial data without any intervention and data after 4 weeks of intervention.

3. Motor Ability

Muscle morphology: Musculoskeletal ultrasound (Konica Minolta, China) will use the L18-4 linear array probe and musculoskeletal mode for the knee joint. The patient will lie supine with a roller under the knee to maintain approximately 30° of knee flexion. The probe will be placed perpendicular to the skin surface at 10 cm above the upper pole of the

patella to measure the thickness and cross-sectional area of the rectus femoris, using ample coupling gel and minimal pressure to reduce soft tissue compression.

Knee extension strength: An isokinetic muscle strength testing system (Guangzhou Yikang, China) will measure peak torque (PT) of knee extension. The patient will sit, and the isokinetic knee joint accessory will be configured. After 5 warm-up repetitions, the patient will perform maximum concentric knee extension and flexion movements at an angular velocity of 60°/s, repeated 5 times. The maximum knee extension peak torque will be recorded in N·m, representing the maximum muscle contraction force.

Motor function: The Fugl-Meyer Motor Assessment for Lower Limbs (FMA-LL) will be used to assess lower limb motor function before and after the intervention, including reflex movements of the lower limbs in the supine position, flexor synergy, extensor resistance movements; combined synergy in the sitting position; isolated movements in the standing position; normal reflexes in the sitting position; and coordination/speed in the supine position. There are 7 major items, totaling 34 minor items, with each minor item scored 1 point, totaling 34 points. A higher score indicates better lower limb functional recovery.

VII. Efficacy Evaluation Criteria

Efficacy evaluation indicators: 1. Brain activation level: Local hemoglobin concentration in the cerebral cortex. 2. Muscle performance: Ultrasound rectus femoris cross-sectional area indicates muscle mass changes; isokinetic muscle strength peak torque indicates muscle explosive power; 3. Physical motor function: Fugl-Meyer Assessment for lower limbs.

Safety evaluation: Incidence of adverse cardiovascular events during rehabilitation. 1) Incidence of skin damage at the cuff site: Nurses will

observe and record the skin condition of the patient's proximal thigh before and after each intervention; 2) Incidence of deep vein thrombosis: Researchers will perform ultrasound evaluation on the day of discharge.

VIII. Observation of Adverse Events

Serious adverse events include secondary stroke or transient ischemic attack, acute myocardial infarction, clinically significant arrhythmia, fracture, pulmonary embolism, and death. Non-serious adverse events include falls, severe pain or muscle soreness, excessively high or low blood pressure, asymptomatic myocardial ischemia, and dizziness. Adverse events will be recorded immediately and reported to the responsible physician, with appropriate emergency measures taken.

IX. Quality Control and Assurance

The members of this research team possess the technical expertise necessary to ensure the smooth implementation of this study. The Neurological Rehabilitation Department of Lianyungang First People's Hospital, where the research team is based, is equipped with the primary facilities and conditions required for this experiment, providing a solid technical foundation. The department has a sufficient number of stroke patients, ensuring the continuous progress of the trial. All functional assessments will be conducted using a blinded method to ensure the scientific rigor and accuracy of the evaluation and treatment. Patients and their families will be informed of relevant knowledge and precautions to ensure participant compliance. The research team is composed of young and talented members with a reasonable structure, covering all the skills required for this project. The team works collaboratively, with each member fulfilling their responsibilities, ensuring the smooth progress of the work.

X. Data Safety Monitoring

The clinical study will develop a corresponding data safety

monitoring plan based on the level of risk. All adverse events will be recorded in detail, appropriately managed, and tracked until they are resolved or the condition stabilizes. Serious adverse events and unexpected incidents will be reported to the ethics committee, relevant authorities, sponsors, and drug regulatory departments in a timely manner as required. The principal investigator will regularly review all adverse events cumulatively and, if necessary, convene researcher meetings to assess the risks and benefits of the study. In double-blind trials, emergency unblinding may be conducted if necessary to ensure the safety and rights of participants. For studies with risks greater than minimal, independent data monitors will be arranged to oversee the research data. High-risk studies will establish an independent data safety monitoring committee to review cumulative safety and efficacy data and make recommendations on whether the study should continue.

XI. Statistical Analysis

Data characteristics will be expressed as mean (standard deviation), median (interquartile range), or percentage. The Shapiro-Wilk test and Levene's test will be used to assess the normality and homogeneity of variance of the data. For data that follow a normal distribution, one-way ANOVA will be used to test differences; otherwise, the Kruskal-Wallis rank-sum test will be used. Chi-square tests will be used for categorical data. Two-way ANOVA will be used to evaluate the main and interaction effects of group and region of interest on HbOmean. If significant, LSD post-hoc tests will be conducted. All data will be analyzed using SPSS 26.0, with a significance level set at $P < 0.05$. Pearson correlation analysis will be used to assess the correlation between cortical HbO and motor performance.

XII. Ethical Considerations in Clinical Research

The clinical research will adhere to the Declaration of Helsinki and

other relevant regulations of the World Medical Association. Before the study begins, the trial protocol must be approved by the ethics committee. Prior to enrolling each participant, the researcher is responsible for providing the participant or their representative with a complete and comprehensive explanation of the study's purpose, procedures, and potential risks. Written informed consent must be obtained, and participants should be informed of their right to withdraw from the study at any time. The informed consent form will be retained as part of the clinical research documentation for future reference. Throughout the research process, the personal privacy and data confidentiality of participants will be protected.

XIII. Research Timeline

December 2023 – June 2024: Participant screening and grouping.

July 2024 – December 2024: Intervention implementation and data collection.

January 2025 – June 2025: Intervention implementation and data collection.

July 2025 – December 2025: Collection and organization of clinical trial data.

January 2026 – June 2026: Completion of all clinical trial cases.

July 2026 – December 2026: Statistical analysis, paper writing, and application for scientific and technological progress awards.

XIV. Project Team Members

<table>
<tr><td rowspan="2">Project Leader</td><td>Name</td><td colspan="2">Zhu Yonggang</td><td>Gender</td><td>Male</td><td colspan="2">Date of Birth</td><td colspan="2">March 3, 1976</td></tr>
<tr><td>Professional Title</td><td colspan="2">Senior</td><td>Degree</td><td>Master of Medicine</td><td colspan="2">Specialization</td><td colspan="2">Clinical Medicine</td></tr>
<tr><td>Project Team</td><td>Total Members</td><td>Senior</td><td>Intermediate</td><td>Junior</td><td>Postdoctoral</td><td>PhD</td><td>Master</td><td colspan="2">Support Staff</td></tr>
</table>

	6	1	3	3		1	3	

	Name	Age	Specialization	Professional Title	Task Assignment	Training Received	Training Period
Main Members of the Project Team	Zhu Yonggang	47	Clinical Medicine	Senior	Topic Selection and Review	Yes	2023.08-2023.10
	Bai Xinyu	41	Clinical Medicine	Intermediate	Design and Paper Writing	Yes	2023.08-2023.10
	Zhang Yunyun	27	Rehabilitation Medicine and Physiotherapy	Junior	Data Analysis, Paper Writing	Yes	2023.08-2023.10
	Cao Weiwei	35	Rehabilitation Therapy	Intermediate	Participant Recruitment	Yes	2023.08-2023.10
	Xie Yang	29	Rehabilitation Therapy	Intermediate	Data Collection	Yes	2023.08-2023.10
	Yu Jie	26	Rehabilitation Therapy	Junior	Trial Implementation	Yes	2023.08-2023.10
	Jiang Ye	28	Rehabilitation Therapy	Junior	Trial Implementation	Yes	2023.08-2023.10

XV. References

[1] Global, regional, and national burden of stroke and its risk factors, 1990-2019: a systematic analysis for the Global Burden of Disease Study 2019 [J]. Lancet Neurol, 2021, 20(10): 795-820.

[2] Kelly-Hayes M, Beiser A, Kase CS, et al. The influence of gender and age on disability following ischemic stroke: the Framingham study [J]. J Stroke Cerebrovasc Dis, 2003, 12(3): 119-126.

[3] Harris ML, Polkey MI, Bath PM, et al. Quadriceps muscle weakness following acute hemiplegic stroke [J]. Clin Rehabil, 2001, 15(3): 274-281.

[4] Cruz-Jentoft AJ, Bahat G, Bauer J, et al. Sarcopenia: revised European consensus on definition and diagnosis [J]. Age Ageing, 2019, 48(1): 16-31.

[5] Liu Q-Q, Xie W-Q, Luo Y-X, et al. High Intensity Interval Training: A Potential Method for Treating Sarcopenia [J]. Clin Interv Aging, 2022, 17: 857-872.

[6] Chang H, Yan J, Lu G, et al. Muscle strength adaptation between high-load resistance training versus low-load blood flow restriction training with different cuff pressure characteristics: a systematic review and meta-analysis [J]. Front Physiol, 2023, 14: 1244292.

[7] Davids CJ, Roberts LA, Bjørnsen T, et al. Where Does Blood Flow Restriction Fit in the Toolbox of Athletic Development? A Narrative Review of the Proposed Mechanisms and Potential Applications [J]. Sports Med, 2023.

[8] Zhang X-Z, Xie W-Q, Chen L, et al. Blood Flow Restriction Training for the Intervention of Sarcopenia: Current Stage and Future Perspective [J]. Front Med (Lausanne), 2022, 9: 894996.

[9] Lamberti N, Straudi S, Donadi M, et al. Effectiveness of blood flow-restricted slow walking on mobility in severe multiple sclerosis: A pilot randomized trial [J]. Scand J Med Sci Sports, 2020, 30(10): 1999-2009.

[10] Gorgey AS, Timmons MK, Dolbow DR, et al. Electrical stimulation and blood flow restriction increase wrist extensor cross-sectional area and flow meditated dilatation following spinal cord injury [J]. Eur J Appl Physiol, 2016, 116(6): 1231-1244.

[11] Freitas EDS, Miller RM, Heishman AD, et al. The perceptual responses of individuals with multiple sclerosis to blood flow restriction versus traditional resistance exercise [J]. Physiol Behav, 2021, 229: 113219.

[12] Du X, Chen W, Zhan N, et al. The effects of low-intensity resistance training with or without blood flow restriction on serum BDNF, VEGF and perception in patients with post-stroke depression [J]. Neuro Endocrinol Lett, 2021, 42(4):

229-235.

[13] Zheng G, Zhou W, Xia R, et al. Aerobic Exercises for Cognition Rehabilitation following Stroke: A Systematic Review [J]. J Stroke Cerebrovasc Dis, 2016, 25(11): 2780-2789.
