# Supplementary material for: Cerebral cortical activation and muscle performance during blood flow restriction training after ischemic stroke: A randomised functional near-infrared spectroscopy study
Source: PLoS One. 2025 Oct 9;20(10):e0334123. doi: 10.1371/journal.pone.0334123 (PMC12510505; doi:10.1371/journal.pone.0334123)
Supplement: S1 Table — (DOCX) [file pone.0334123.s003.docx]

**Table S3.** Results of multiple comparisons (LSD post-hoc tests) for group and region of interest effects.

| **Comparison Type** | **Comparison** | **HBO_1_** | |  | **ΔHBO** | |
| --- | --- | --- | --- | --- | --- | --- |
|  |  | **Mean Difference [95% CI]** | **p-value** |  | **Mean Difference [95% CI]** | **p-value** |
| **Group Comparisons** | BFR vs LL | 0.07[0.02, 0.11] | <0.003** |  | 0.05[0.01-0.09] | 0.010** |
|  | BFR vs HL | 0.02[-0.03, 0.06] | 0.511 |  | 0.01[-0.03-0.04] | 0.711 |
|  | LL vs HL | 0.05[-0.76, -0.14] | 0.020* |  | 0.04[0.0-0.08] | 0.028* |
| **ROI Comparisons** | LDLPFC vs LM1 | 0.13[0.07-0.19] | <0.001 |  | 0.0[-0.06-0.06] | 0.997 |
|  | LDLPFC vs LPMASMC | 0.14[0.08-0.20] | <0.001 |  | -0.01[-0.06-0.05] | 0.805 |
|  | LDLPFC vs RDLPFC | 0.05[-0.01, 0.11] | 0.122 |  | 0.03[-0.03-0.08] | 0.379 |
|  | LM1 vs LPMASMC | 0.01[-0.05-0.07] | 0.759 |  | 0.01[-0.06-0.05] | 0.802 |
|  | LM1 vs RM1 | 0.09[0.02, 0.15] | 0.008** |  | 0.06[0.0-0.12] | 0.034* |
|  | LPMASMC vs  RPMASMC | 0.06[0, 0.13] | 0.060 |  | 0.06[0.01-0.12] | 0.031* |
|  | RDLPFC vs RM1 | 0.17[0.10-0.23] | <0.001 |  | 0.04[-0.02-0.09] | 0.213 |
|  | RDLPFC vs RPMASMC | 0.15[0.09-0.22] | <0.001 |  | 0.04[-0.01-0.10] | 0.125 |
|  | RM1 vs RPMASMC | 0.02[-0.05-0.08] | 0.640 |  | 0.01[-0.05-0.06] | 0.772 |
