# Supplementary material for: Cerebral cortical activation and muscle performance during blood flow restriction training after ischemic stroke: A randomised functional near-infrared spectroscopy study
Source: PLoS One. 2025 Oct 9;20(10):e0334123. doi: 10.1371/journal.pone.0334123 (PMC12510505; doi:10.1371/journal.pone.0334123)
Supplement: Attachment — Submitted filename: Response to Reviewers.docx [file pone.0334123.s005.docx]

Dear Editors

We gratefully appreciate the editors and all reviewers for their time spend making positive and constructive comments. Those comments are all valuable and helpful for revising and improving our article entitled “**Cerebral cortical activation and muscle performance during blood flow restriction training after ischemic stroke: a randomised functional near-infrared spectroscopy study**” (ID: **PONE-D-25-08906**), as well as the important guiding significance to our research.

We have studied the comments carefully and have made corrections which we hope meet with approval. Point-to-point responses to **academic editor** and **reviewers** are listed below. Revised portions are highlighted in the manuscript.

Thank you and best regards.

**Responses to academic editor (Responses by the author are in blue)**

**Academic editor Comments:**

**1.** When submitting your revision, we need you to address these additional requirements. Please ensure that your manuscript meets PLOS ONE's style requirements, including those for file naming. If any supporting files for review show as item type‘other’please change to item type‘supporting info’as the reviewer does not have access to these ’other’ files.

**Response:** Thank you for your feedback and the opportunity to revise our manuscript. We have carefully addressed the additional requirements outlined in your message. Specifically, we confirm the following:

1. **PLOS ONE Style Requirements:**

The manuscript has been reformatted to fully comply with PLOS ONE’s style guidelines, including references, figure/table labeling, and ethical/legal standards.

All supporting files (figures, tables, supplemental materials) have been checked for formatting consistency.

2. **File Naming Conventions:**

All submission files (manuscript, figures, supplementary materials) have been renamed according to PLOS ONE’s requirements.

3. **Supporting File Item Types**:

Any supporting files previously categorized as “other” in the submission system have been updated to the correct item type “supporting information” to ensure reviewers have full access.

We have double-checked the revised submission to ensure all editorial and stylistic criteria are met. Should further adjustments or clarifications be needed, please don’t hesitate to contact us.

**2.** We note that your Data Availability Statement is currently as follows: [All relevant data are within the manuscript and its Supporting Information files.]

Please confirm at this time whether or not your submission contains all raw data required to replicate the results of your study. Authors must share the “minimal data set” for their submission. PLOS defines the minimal data set to consist of the data required to replicate all study findings reported in the article, as well as related metadata and methods (https://journals.plos.org/plosone/s/data-availability#loc-minimal-data-set-definition).

**Response:** Thank you for your kind remind. We confirm that our submission includes the minimal data set required to replicate all study findings. All raw experimental data, associated metadata, and methodological details are provided within the Supporting Information files.

**3.** PLOS requires an ORCID iD for the corresponding author in Editorial Manager on papers submitted after December 6th, 2016. Please ensure that you have an ORCID iD and that it is validated in Editorial Manager. To do this, go to ‘Update my Information’ (in the upper left-hand corner of the main menu), and click on the Fetch/Validate link next to the ORCID field. This will take you to the ORCID site and allow you to create a new iD or authenticate a pre-existing iD in Editorial Manager.

**Response:** We confirm that the corresponding author’s ORCID iD has been validated in Editorial Manager following the journal’s requirements. The ORCID record is now fully linked and accessible in the system. Thank you for the guidance.

**4.** Please amend either the title on the online submission form (via Edit Submission) or the title in the manuscript so that they are identical.

**Response:** Thank you for your note. We confirm that the manuscript title has been revised to match exactly the title in the online submission system. Minor edits were made during CONSORT 2010 checklist alignment (e.g., clarifying study design), and both versions are now consistent. We apologize for the oversight and appreciate your guidance.

**Responses to Reviewers (Responses by the author are in blue)**

**Reviewer #1:**

**1. Comments**: The sample size calculation with power considerations as well as the statistical analysis plan is consistent from the supplemental protocol to the written manuscript. The analysis elements are routine and applied appropriately for a three group design. They include the Shapiro-Wilk test and Levene's test assess the normality and homogeneity of variance of the data. For data that follow a normal distribution, one-way ANOVA was used to test differences; otherwise, they say that the Kruskal-Wallis rank-sum test will be used. All of the results reporting appears to be normality based. Was there any non parametric reporting or need for such?
**Response:** Thank you so much for your careful review and thoughtful feedback! We sincerely appreciate your attention to detail. Regarding your question, we did use non-parametric methods where assumptions were not met. For example, Time poststroke (**Table 1**) did not follow a normal distribution (Shapiro-Wilk test, p < 0.05), so we applied the Kruskal-Wallis test, as stated in our statistical plan. These results are reported in Section Result (**Line181-182**) of the manuscript. Your insights are invaluable, and we are happy to provide additional details if needed!
**2.** Chi-square tests were used for categorical data. Two-way ANOVA was used to evaluate the main and interaction effects of group and region of interest on HbO mean. If significant, LSD post-hoc tests will be conducted. For any post hoc comparisons with p-values, what was the post hoc test used?
**Response:** Thank you for your advice. For significant main or interaction effects identified in the two-way ANOVA (group × region of interest), LSD test was applied for pairwise multiple comparisons. The validity of this approach was ensured by confirming the homogeneity of variance assumption. Detailed statistical procedures for multiple comparisons are described in the Methods section (**Lines 170-172**); Key comparison results are reported in the Results section (**Lines 189-193** and **Lines 202-206**); Complete numerical outcomes of all pairwise comparisons (including mean differences, p-values, and confidence intervals) are provided in **Supplementary Table S3**.
**3.** Pearson correlation analysis was used to assess the correlation between cortical HbO and motor performance. This reporting was reasonable.
**Response:** Thank you sincerely for your positive feedback and careful evaluation of our analysis!
**4.** The sample size called for 22 per group and 15% dropout rate. Table 1 shows 20 per group. Did all 20 per group complete the analysis in Tables 2 and 3? If not, is there a missing data issue?
**Response:** Thank you for raising this critical point. We clarify as follows:

**1. Sample size validity**: The final analyzed sample (n=20 per group, total n=60) exceeds the pre-specified minimum requirement of 57 subjects stated in the Statistical Power Calculation subsection (Methods, Section **Statistical Analysis**).

**2. Dropout handling transparency**: As noted in the revised Results section: "Missing data due to dropouts were not imputed." Attrition details (e.g., discharge, muscle soreness) are fully documented in Fig 1 (CONSORT flowchart) and Table 1 (baseline data of analyzed cohorts)

**3. Analysis consistency**: All results in Tables 2-3 strictly reflect complete cases (n=20 per group). No intention-to-treat (ITT) analysis was performed given the lack of post-dropout endpoint data, aligning with our pre-registered complete-case protocol.

We appreciate the opportunity to strengthen methodological clarity.
5. Why is there no p-value for group comparisons (pre-post) in Table 3? It is not entirely clear in the paragraph discussing the results of Table 3.
**Response:** We sincerely appreciate your careful review of the statistical reporting. We have made the following clarifications and revisions to address this concern.

The asterisk (*) in Table 3 denotes significant within-group pre-post differences (P<0.05), while the hash symbol (#) indicates significant between-group differences (P<0.05) after intervention. These definitions are now explicitly stated in the table footnote (see revised Table 3 caption).

In the revised Results section (**Lines 226-234**), we have added detailed statistical descriptors: “PT and FMLE scores significantly improved post-intervention in BFR and HL groups (all *P*<0.05 vs. pre-intervention), with CSA showed no significant in either the BFR group (*P*=0.296, pre- vs. post-intervention) or the HL group (*P*=0.144). The LL group showed no significant changes in PT, FMLE, or CSA (all *P*>0.05 pre- vs. post-intervention). Compared to the LL group, the HL group demonstrated significantly greater improvements in PT (*P*<0.001) and FMLE (*P*=0.023), while the BFR group showed enhanced PT only (*P*<0.001 vs. LL). No significant differences in CSA were observed between HL, BFR, and LL groups (*P*> 0.05). Furthermore, direct comparisons between the BFR and HL groups revealed no statistically significant differences in CSA, PT, or FMLE scores (all *P*>0.05). Complete statistical results are presented in Table 3.

6. The investigators have clearly listed the limitations in the discussion. Given the detection time points schematic in the protocol, why is there not a time factor for data (re-measurement) in the overall ANOVA?

**Response:** Thank you for raising this critical methodological point. Below are our detailed responses regarding the temporal factor analysis:

1. Rationale for not applying repeated-measures ANOVA
   Hemodynamic data were collected only at two time points (baseline: first training session; endpoint: last training session). As repeated-measures ANOVA generally requires ≥3 time points to adequately model temporal trends and within-subject variability, we adopted the following alternative strategies to assess time effects while avoiding overinterpretation of limited longitudinal data:
2. Alternative analytical approaches for temporal effects
   (1) Baseline brain activation: Initial neural responses were analyzed using two-way ANOVA (Group × ROI) at the first training session;
   (2) Delta-value analysis: We computed per-subject ΔHbO (last session minus first session) and examined group/ROI effects on these delta values via the same ANOVA framework;
   (3) Clinical correlation validation: Pearson/Spearman correlations between ΔHbO and concurrent changes in muscle mass/strength/performance were performed (Fig 5), establishing temporal coherence between neuroadaptation and functional outcomes.

We fully agree that the two-timepoint design limits granular temporal modeling. Future studies will incorporate intermediate assessments to enable more sophisticated longitudinal analyses.

**Reviewer #2 Comments**

1. The objective sentence of the manuscript must be strengthened and must give more context about the importance and possible beneficial effects of the study into the related literature.

**Response:** Thank you for emphasizing the need to contextualize our study's objectives within the broader literature. We have substantially strengthened the objective statement and background rationale in the Introduction (**Lines 25-55**), with objective sentence revisions outlined below: To resolve the critical knowledge gaps in BFRT-induced neuroplasticity, we conducted a randomized controlled trial integrating real-time fNIRS with muscle strength-structure-function profiling. We aimed to: (a) Compare acute-phase cortical activation (prefrontal-motor HbO) across BFRT (30%1RM+occlusion), HLRT (80%1RM), and LLRT (30%1RM); (b) Investigate temporal associations between HbO changes and improvements in muscle strength, hypertrophy , and motor function; (c) Validate if BFRT induces HLRT-equivalent neuroplasticity with 50% lower mechanical loads, prioritizing central drive over peripheral adaptations. These revisions explicitly anchor our objectives within unresolved theoretical debates (e.g., central-peripheral interplay) and unmet clinical needs (e.g., load-intolerant patients). We appreciate your guidance in elevating the manuscript's scholarly impact.

2. At the end of the conclusions section, please provide point to point recommendations coaches, patients and sport scientists about the potential applications of your results to improve the quality of exercise sessions in ischemic stroke patients.

**Response:** Thank you for highlighting the importance of translational applications. We have revised the Conclusions section (Lines **379-386**) to include targeted recommendations for stakeholders, as detailed below: For coaches and therapists, implementing BFRT (30% 1RM with occlusion) or HIRT (80% 1RM) during early rehabilitation can effectively enhance neural drive, while real-time fNIRS monitoring allows personalized adjustment of exercise intensity. For patients, supervised BFRT/HIRT combined with functional tasks promotes synergistic neural-muscular improvements, with progress tracked through both wearable fNIRS-based brain activation and strength metrics. For researchers, optimizing BFRT protocols to amplify long-term neuroplasticity and muscle hypertrophy, alongside validating HbO as a rehabilitation efficacy biomarker, will advance precision rehabilitation strategies.

We tried our best to improve the manuscript and made some changes in the manuscript. We think that our manuscript has greatly improved after revisions, and we would like to submit the revised manuscript for publication in *Plos one.* We appreciate the reviewers’ warm work earnestly and hope that the correction will meet with approval. Once again, thank you very much for your comments and suggestions.

Best wishes,

May 8th, 2025
