# Supplementary material for: Cerebral cortical activation and muscle performance during blood flow restriction training after ischemic stroke: A randomised functional near-infrared spectroscopy study
Source: PLoS One. 2025 Oct 9;20(10):e0334123. doi: 10.1371/journal.pone.0334123 (PMC12510505; doi:10.1371/journal.pone.0334123)
Supplement: Attachment — Submitted filename: Response_to_Reviewers_auresp_2.docx [file pone.0334123.s006.docx]

Dear Editors

We gratefully appreciate the editors and all reviewers for their time spend making positive and constructive comments. Those comments are all valuable and helpful for revising and improving our article entitled “**Cerebral cortical activation and muscle performance during blood flow restriction training after ischemic stroke: a randomised functional near-infrared spectroscopy study**” (ID: **PONE-D-25-08906R1**), as well as the important guiding significance to our research.

We have studied the comments carefully and have made corrections which we hope meet with approval. Point-to-point responses to **reviewers** are listed below. Revised portions are highlighted in the manuscript.

Thank you and best regards.

**Responses to Reviewers (Responses by the author are in blue)**

**Reviewer #3:**

**1. Comments**: The title entered in Editorial Manager is still in Chinese, whereas the English title appears on the manuscript title page. Update the submission-system title (currently in Chinese) to match the English manuscript title to avoid downstream indexing errors.

**Response:** We sincerely apologize for this administrative oversight. The English manuscript title has now been updated in Editorial Manager to match the title page (current Chinese title removed). This correction ensures consistency across all submission records to prevent downstream indexing issues. Thank you for catching this critical detail.

**2.**  **Comments**: There are some typographic errors, data-availability formalities as well as the abbreviation and acronym definitions. Please try to find all, and fix them.
- Both HLRT and HIRT are used for high-load training, choose one acronym and apply consistently.)
- “HBO” appears several times; accepted symbol is HbO, uniformly replace with HbO.
- Run spell-check to catch “frist”.
- Add “(fNIRS)” in the Abstract, use the acronym after introduced at first for consistency.
- Standardise percentage formatting (use either 70 % or 70%, not both)
**Response:** We thank the reviewer for these critical technical corrections. Throughout the manuscript, we have: standardized the acronym to HLRT (removing all 'HIRT' instances); corrected 'HBO' to 'HbO' for oxyhemoglobin notation; fixed typographical errors (e.g., 'frist'→'first') via full spell-check; added '(fNIRS)' after the first abstract mention; normalized percentage formatting to 70%. These updates ensure terminological precision and formatting consistency across the submission.

**Reviewer #4**

**1. Comments**：The introduction mostly references general stroke and resistance training literature but could benefit from deeper integration of BFRT-specific neuroimaging studies, especially in clinical populations.
Integrate 1–2 key studies that examined BFRT’s cortical effects (e.g., via TMS, EEG, fMRI) in healthy or stroke subjects to better frame the study’s novelty.

**Response:** We thank the reviewer for highlighting the need for deeper integration of BFRT-specific neuroimaging literature. In response, we have incorporated three pivotal studies examining BFRT's cortical effects in the Introduction (Lines 41-46): Crucially, BFRT demonstrates amplified cortical responses in healthy individuals, with TMS showing significantly greater corticospinal excitability during upper- or lower-limb BFRT versus low load (LL) RT[12, 13]. Specifically, functional near-infrared spectroscopy (fNIRS) reveals optimal motor cortex activation during squatting with moderate-pressure BFRT (250 mmHg), outperforming both non-restricted (0 mmHg) and alternative pressure conditions (150 and 300 mmHg)[7].

**2. Comments**：Using a fixed pressure (200 mmHg) for all participants may lead to uneven BFRT stimulus, especially considering individual differences in limb size and vascular resistance.

**Response**: We appreciate the reviewer's valid concern regarding individualized pressure prescription. Our pressure selection rationale is now explicitly stated in the Methods section (Lines 96-99): Previous studies in healthy subjects have indicated that a pressure of 250 mmHg is optimal for achieving the greatest degree of cerebral activation[7]. Considering that circulation may be impaired in stroke patients, we set the pressure value at 80% (200 mmHg). Furthermore, we address generalizability limitations in the Limitations section (Line 371-374): Current arterial occlusion pressure (AOP) recommendations span 40-80%[51], while applied pressures range widely (50-230 mmHg across literature). Our use of standardized 200 mmHg pressure - implemented for safety and operational feasibility in stroke patients, which may impact generalizability.
**3. Comments**：Clarify that short-term strength gains likely reflect neural adaptation rather than hypertrophy.

**Response**: We sincerely appreciate the reviewer's insightful comment regarding the mechanistic interpretation of short-term strength gains. We fully agree that early-phase improvements in strength are predominantly driven by neural adaptations rather than hypertrophic changes. To explicitly address this point:

**Discussion Reinforcement:**
As noted in our Discussion (lines 261-322), we have consistently emphasized the role of **acute and chronic neural adaptations** as the primary mechanism underpinning early strength gains.

Following the reviewer’s suggestion, we have added the following statement to the Conclusions section (Lines 381-384) to crystallize this distinction：The present study highlights the superiority of BFRT and HLRT over LLRT alone for the development of central activation and muscle performance through neural adaptations rather than hypertrophic changes. Training programs should therefore prioritize neuromodulatory stimuli via targeted exercise variable manipulation.
**4. Comments**：Include ITT as sensitivity analysis in future studies or at least discuss this limitation.

**Response**: We sincerely thank the reviewer for raising this important point regarding the use of intention-to-treat (ITT) analysis. As the reviewer suggested, we have explicitly discussed this limitation in the revised manuscript (Line374-377)：Third, our analytical approach followed per-protocol rather than intention-to-treat (ITT) principles. Future investigations should incorporate ITT analysis to evaluate robustness against attrition bias and maintain randomization integrity.

We confirm that all figure files have been successfully processed through the PACE tool prior to resubmission.

We tried our best to improve the manuscript and made some changes in the manuscript. We think that our manuscript has greatly improved after revisions, and we would like to submit the revised manuscript for publication in *Plos one.* We appreciate the reviewers’ warm work earnestly and hope that the correction will meet with approval. Once again, thank you very much for your comments and suggestions.

Best wishes,

July 2th, 2025
